# Cytotoxicity and inflammatory potential of two *Pseudomonas mosselii* strains isolated from clinical samples of hospitalized patients

**DOI:** 10.1186/1471-2180-13-123

**Published:** 2013-05-29

**Authors:** Charlène Leneveu-Jenvrin, Amar Madi, Emeline Bouffartigues, Kelly Biaggini, Marc Feuilloley, Sylvie Chevalier, Nathalie Connil

**Affiliations:** 1 Laboratoire de Microbiologie Signaux et Microenvironnement (LMSM) EA 4312, Université de Rouen, Normandie Université, 55 rue Saint-Germain, Evreux F-27000, France

**Keywords:** *Pseudomonas mosselii*, Cytotoxicity, Inflammation, F-actin cytoskeleton damage

## Abstract

**Background:**

The genus *Pseudomonas* includes a heterogeneous set of microorganisms that can be isolated from many different niches and nearly 100 different strains have been described. The best characterized bacterium is *Pseudomonas aeruginosa* which is the primary agent of opportunistic infection in humans, causing both acute and chronic infections. Other species like *fluorescens*, *putida* or *mosselii* have been sporadically isolated from hospitalized patients but their association with the pathology often remains unclear.

**Results:**

This study focuses on the cytotoxicity and inflammatory potential of two strains of *Pseudomonas mosselii* (ATCC BAA-99 and MFY161) that were recently isolated from clinical samples of hospitalized patients. The behavior of these bacteria was compared to that of the well-known opportunistic pathogen *P. aeruginosa* PAO1. We found that *P. mosselii* ATCC BAA-99 and MFY161 are cytotoxic towards Caco-2/TC7 cells, have low invasive capacity, induce secretion of human β-defensin 2 (HBD-2), alter the epithelial permeability of differentiated cells and damage the F-actin cytoskeleton.

**Conclusions:**

These data bring new insights into *P. mosselii* virulence, since this bacterium has often been neglected due to its rare occurrence in hospital.

## Background

The genus *Pseudomonas* is one of the most diverse and ecologically significant bacterial groups on the planet. The large size and the plasticity of their genome explain at least partly their ability to cope with different forms of stresses (physical, chemical or antimicrobial agents) resulting in their widespread distribution [1]. The genus *Pseudomonas* includes more than 100 species, a number that is increasing in time [2]. Nearly each year, a new species is indeed discovered, like *P. duriflava, P. batumici* or *P. litoralis* for example, isolated from a desert soil [3], the Caucasus Black sea coast [4] or from Mediterranean seawater [5], respectively. Due to its heterogeneity, the genus *Pseudomonas* has undergone numerous taxonomic changes depending on the criteria employed for their definition and delineation: phenotypic, physiologic or metabolic characteristics, siderotyping, phylogeny based on 16S rRNA and/or “housekeeping” genes, analysis of 16S-23S rRNA intergenic spacers (ITS) or the use of functional and ecological genetic markers such as *oprF*, *oprD* or *gacA*[2,6-8].

*P. aeruginosa* is by far the most studied species in the genus *Pseudomonas*. It is an opportunistic pathogen that provokes nosocomial infection and causes severe acute and chronic infections either in healthy or in immunocompromised individuals [9]. Other *Pseudomonas* species have been suspected in human infections [2]. For example, the very common environmental contaminant *P. fluorescens* has also been associated to various clinical cases [10-14]. This bacterium may particularly colonize the airways, the urinary tract and blood of immunocompromised patients. Recently, some *P. fluorescens* strains were found to behave as human pathogens, since they have a high hemolytic activity and dispose of a complete type three secretion system arsenal [15-18].

*P. mosselii* is a novel species, which has been characterized in 2002 [19]. It has been linked to *P. putida* clinical strains using 16SrDNA, *oprF* and *oprD* as markers for phylogeny-based studies [7,8]. In 2009, McLellan and Partridge [20] presented a case of prosthetic valve endocarditis caused by *P. mosselii*. These authors proposed that *P. mosselii* should be regarded as a potential pathogen.

In a previous study, we have found that *P. mosselii* strains were able to adhere and to display a necrotic potential on rat glial cells [21]. To get further insights into *P. mosselii* virulence, we investigate in the present work the cytotoxicity and proinflammatory effects of two clinical strains of *P. mosselii* (ATCC BAA-99 and MFY161) on Caco2/TC7 cells, the transepithelial permeability of Caco2/TC7 monolayers and the actin network. The behavior of these bacteria was compared to that of the well-known opportunistic pathogen *P. aeruginosa* PAO1.

## Results

### Cytotoxicity assay

**Figure 1:**
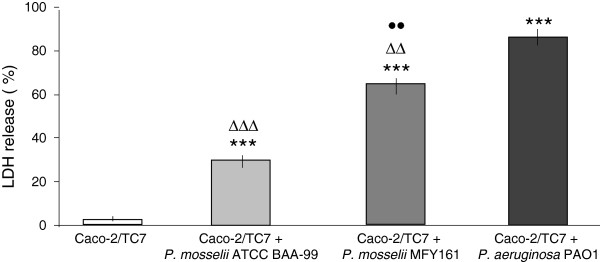
**Cytotoxic effects of *P. mosselii *ATCC BAA-99, *P. mosselii *MFY161 and *P. aeruginosa *PAO1 on Caco-2/TC7 cells.** Cytotoxicity was determined by LDH release assay after 24 h of infection. Results were calculated as the mean values (+/−SEM) of three independent experiments. *** P < 0.001 versus uninfected Caco-2/TC7 cells, ∆∆∆ P < 0.001 versus *P. aeruginosa* PAO1*,* ∆∆ P < 0.01 versus *P. aeruginosa* PAO1, •• P < 0.01 versus *P. mosselii* ATCC BAA-99.

The cytotoxic effect of *P. mosselii* ATCC BAA-99 and MFY161 on Caco-2/TC7 cells was determined by quantification of lactate dehydrogenase (LDH) released in culture medium (Figure 1). The results show that *P. mosselii* ATCC BAA-99 was the less cytotoxic strain with only 30.1 +/−0.1% of cell lysis after 24 h of infection. *P. mosselii* MFY161 exhibited a cytotoxic activity reaching 64.5 +/−0.1% of lysis and the cytotoxic activity of *P. aeruginosa* PAO1 was higher with 85.6 +/−0.2% of lysis. Enumeration of *P. mosselii* ATCC BAA-99 (5 × 10^8^ CFU.mL^-1^), *P. mosselii* MFY161 (4.8 × 10^8^ CFU.mL^-1^) and *P. aeruginosa* PAO1 (4.9 × 10^8^ CFU.mL^-1^), at the end of the infection period showed that higher cytotoxicity was not due to bacterial overgrowth.

### Bacterial invasion assay

**Figure 2:**
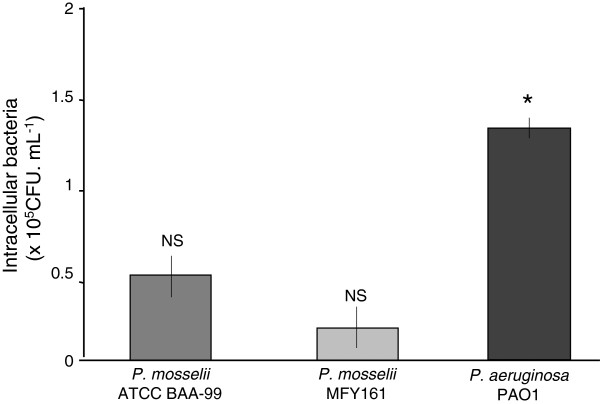
**Invasive capacity of *P. mosselii *ATCC BAA-99, *P. mosselii *MFY161 and *P. aeruginosa *PAO1.** 4 h after infection of Caco-2/TC7 cells with the bacteria, extracellular germs were killed by gentamicin. Cells were lysed and the intracellular bacteria were enumerated by plating onto nutrient agar medium. Results were calculated as the mean values (+/−SEM) of three independent experiments. * P < 0.05 versus *P. mosselii* ATCC BAA-99 and *P. mosselii* MFY161, NS not significant between *P. mosselii* ATCC BAA-99 and *P. mosselii* MFY161.

The capacity of *P. mosselii* ATCC BAA-99 and MFY161 to enter Caco-2/TC7 cells has been investigated using the gentamicin exclusion test (Figure 2). The results show that the two *P. mosselii* strains studied can have an invasive behavior with 0.5 +/−0.2 × 10^5^ and 0.2 +/−0.2 × 10^5^ CFU.mL^-1^ detected intracellularly for *P. mosselii* ATCC BAA-99 and MFY161, respectively. The invasive capacity of *P. aeruginosa* PAO1 was significantly higher with 1.4 +/−0.1 × 10^5^ CFU.mL^-1^ that entered Caco-2/TC7 cells.

### Quantification of IL-6, IL-8 and HBD-2 secretion

**Figure 3:**
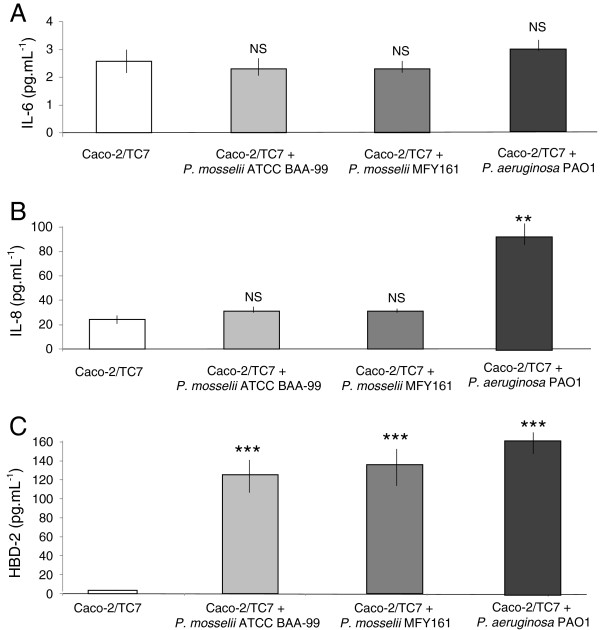
**Proinflammatory effects of *P. mosselii *ATCC BAA-99, *P. mosselii *MFY161 and *P. aeruginosa *PAO1 on Caco-2/TC7 cells.** IL-6 and IL-8 cytokines, and HBD-2 were measured in Caco-2/TC7 cells supernatant after 24 h of infection. Results were calculated as the mean values (+/−SEM) of three independent experiments. *** P < 0.001 versus uninfected Caco-2/TC7 cells, ** P < 0.01 versus uninfected Caco-2/TC7 cells, NS not significant.

The bacterial proinflammatory effect of *P. mosselii* ATCC BAA-99, *P. mosselii* MFY161 and *P. aeruginosa* PAO1 was assessed by measuring IL-6 and IL-8 secretion in Caco-2/TC7 after 24 h of infection. The results show that the two strains of *P. mosselii* studied did not induce significant stimulation of IL-6 (Figure 3A) and IL-8 (Figure 3B) secretion in Caco-2/TC7 compared to uninfected cells. On the contrary, the infection of Caco-2/TC7 cells with *P. aeruginosa* PAO1 led to a major secretion of IL-8 with 92 +/−13 pg.mL^-1^ (Figure 3B).

The human β-defensin-2 (HBD-2) was also quantified in Caco-2/TC7 cells supernatant. The results show that the two strains of *P. mosselii* were able to induce HBD-2 secretion by Caco-2/TC7 cells (Figure 3C). Infection with *P. mosselii* ATCC BAA-99 and MFY161 strains led to a major increase of HBD-2 production by Caco-2/TC7 with 125 +/−26 pg.mL^-1^ and 136 +/−31 pg.mL^-1^, respectively, compared to the 4 +/−2 pg.mL^-1^ basal secretion of HBD-2 in uninfected cells. The induction of HBD-2 by the two *P. mosselii* strains was almost similar to that obtained with *P. aeruginosa* PAO1 (165 +/−14 pg.mL^-1^).

### Transepithelial electrical resistance measurements

**Figure 4:**
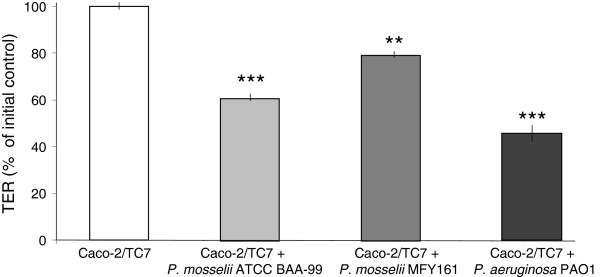
**Effects of *P. mosselii *ATCC BAA-99, *P. mosselii *MFY161 and *P. aeruginosa *PAO1 on the transepithelial electrical resistance of Caco-2/TC7 cells.** Differentiated Caco-2/TC7 cells were infected for 24 h. The TER was expressed as percentages of the initial control TER measured across each individual cell monolayer at the onset of the experiment. Results are the mean values (+/−SEM) of three independent experiments. *** P < 0.001 versus uninfected Caco-2/TC7 cells, ** P < 0.01 versus uninfected Caco-2/TC7 cells.

The effect of the bacteria on epithelial permeability was evaluated by measuring the TER across differentiated Caco-2/TC7 monolayers. TER values were measured at the onset of the experiment and at times 3, 6, 9 and 24 h. Up to 9 h after the beginning of the experiment, the TER values of the infected monolayers remained unchanged (data not shown). After 24 h of infection, the TER values of the monolayers exposed to the bacteria were significantly decreased (Figure 4). The decrease of TER induced by *P. mosselii* MFY161 was 20.8 +/−4.7% compared to uninfected control cells whereas *P. mosselii* ATCC BAA-99 led to a decrease of TER reaching 39 +/− 3.2% and *P. aeruginosa* PAO1 provoked a deeper decrease of the TER value (55.8 +/−5.3%). These falls in TER cannot be attributed to damages provoked by acidification of the medium since the pH of the medium remained constant over the studies.

### Actin visualisation

**Figure 5:**
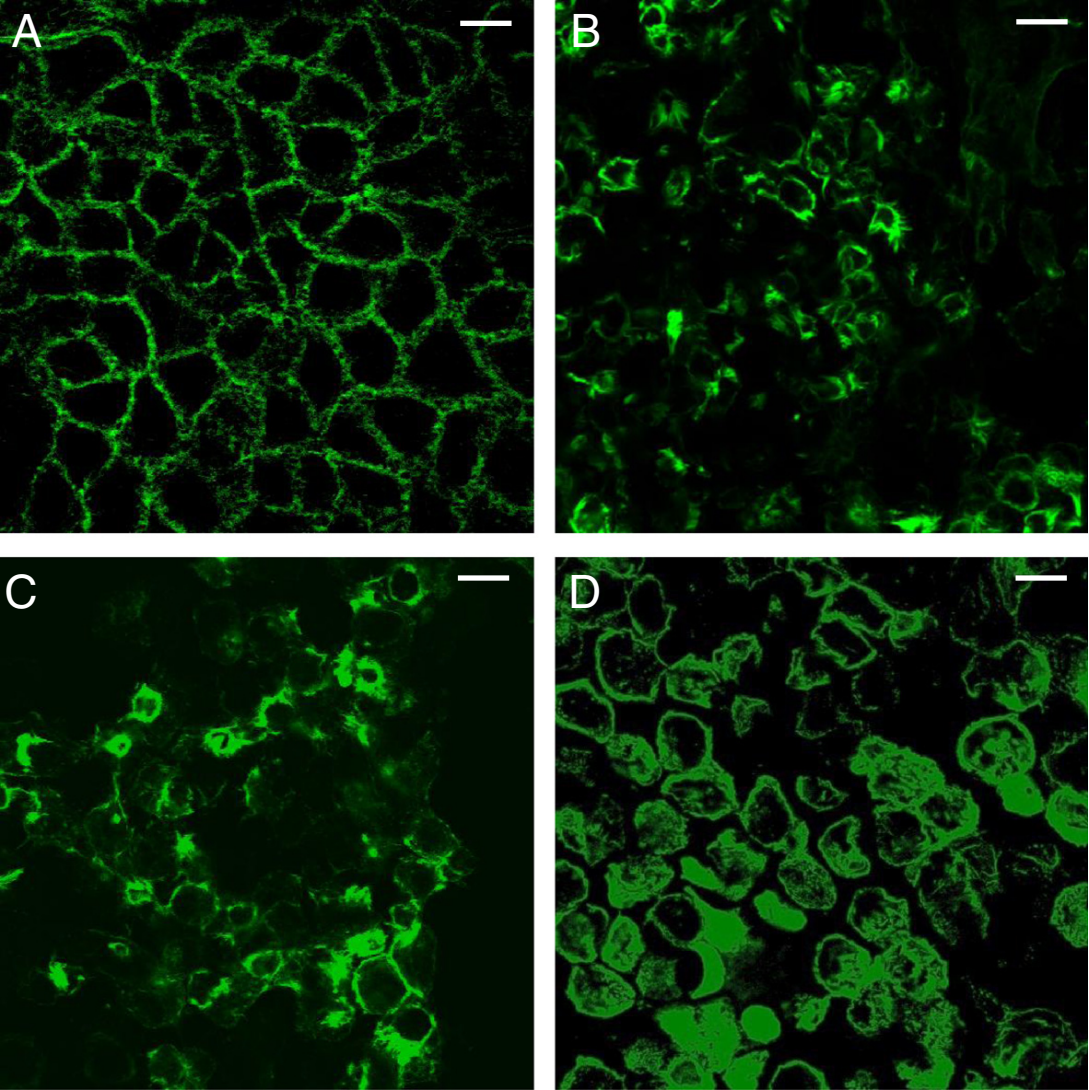
**Effects of *P. mosselii *ATCC BAA-99, *P. mosselii *MFY161 and *P. aeruginosa *PAO1 on the F-actin cytoskeleton of Caco-2/TC7 cells.** Differentiated Caco-2/TC7 cells were infected for 24 h. F-actin was stained and examined using a confocal laser scanning microscope. (**A**) Uninfected cells, (**B**) infection by *P. mosselii* ATCC BAA-99, (**C**) infection by *P. mosselii* MFY161, (**D**) infection by *P*. *aeruginosa* PAO1. Scale bar 100 μm.

The effect of *P. mosselii* ATCC BAA-99 and MFY161 on the organization of the sub-membrane F-actin microfilament network was studied and compared to that of *P. aeruginosa* PAO1. Whereas the staining pattern of untreated Caco-2/TC7 cells showed a continuous fine meshwork of microfilaments lining the cell border (Figure 5), the cells exposed for 24 h with *P. mosselii* ATCC BAA-99, *P. mosselii* MFY161 or *P. aeruginosa* PAO1 lost their normal organization. All these bacteria induced a dramatic disruption of F-actin.

## Discussion

*P. mosselii* was formally described as a novel species in 2002 through a polyphasic taxonomic approach including 16SrDNA phylogeny, numerical analysis, DNA–DNA hybridization, thermal stability of DNA–DNA hybrids and siderophore-typing methodology [19]. The several strains of *P. mosselii* described to date were isolated in hospital and some have been suggested as emerging human pathogens [19-21]. Our study aimed at investigating the virulence potential of two of these strains, namely ATCC BAA-99 and MFY161, belonging to the same cluster strongly related to the hospital-isolated *P. putida* on the basis of both *oprD* or *oprF*-linked phylogenies [22]. Although *P. putida* species is mostly known for its huge capacity in degradation of numerous carbon sources [23], some clinical strains have emerged, causing infections in immunosuppressed hosts and patients with invasive medical devices. More recently, *P. putida* has been involved in war wound infection, and should be considered as a potential human pathogen, for a review see Carpenter et al. [24].

In the present study, we further investigated the cytotoxicity of *P. mosselii* ATCC BAA-99 and MFY161 strains, and show that they provoked the lysis of the intestinal epithelial cells Caco-2/TC7, with a major damage obtained after infection with *P. mosselii* MFY161. The cytotoxic levels were lower compared to the well-known opportunistic pathogen *P. aeruginosa* PAO1 but almost similar to those observed for *P. mosselii* strains on rat glial cells [21], and for the clinical strain *P. fluorescens* MFN1032 on Caco-2/TC7 cells [17]. The gentamicin exclusion test showed that *P. mosselii* ATCC BAA-99 and MFY161 can enter Caco-2/TC7 cells. The invasion capacity of the two *P. mosselii* strains studied was similar and lower than that of the pathogen *P. aeruginosa* PAO1.

The bacterial proinflammatory effect of *P. mosselii* ATCC BAA-99 and MFY161 was then assessed by measuring the secretion of IL-6 and IL-8 cytokines in Caco-2/TC7 after 24 h of infection. The results showed that the two strains did not induce the production of these proinflammatory cytokines. We hypothesize that this may serve as a strategy for *P. mosselii* to escape the immune system. However, *P. mosselii* ATCC BAA-99 and MFY161were found to strongly increase the secretion of HBD-2. Human beta-defensins are known to play a key role in host defense. In fact, in addition to their potent antimicrobial properties against commensal and pathogenic bacteria [25], beta-defensins were demonstrated to function as multieffector molecules capable of enhancing host defense by recruiting various innate as well as adaptive immune cells to the site of infection. Nevertheless, some pathogens can be resistant to HBD-2 [26] and surprisingly can induce and divert HBD-2 secretion in intestinal epithelial cells to enhance its capacity of virulence [27].

The effect of *P. mosselii* ATCC BAA-99 and MFY161 on epithelial permeability was then evaluated by measuring the TER across differentiated Caco-2/TC7 monolayers. The F-actin cytoskeleton was stained with Alexa-488 phalloïdin and examined using a confocal laser scanning microscope. We observed that the TER of the monolayers exposed to the bacteria was significantly decreased and that the F-actin cytoskeleton was completely broken. Similar results of TER decrease and F-actin disruption were previously observed with many pathogens including *Salmonella typhimurium*, *P. aeruginosa* and *Escherichia coli*[28-30].

Infections caused by multidrug-resistant (MDR) Gram-negative bacilli have become a growing challenge in hospital [31]. In a recent study, Giani et al. [32] suggested that unusual human opportunistic pathogen like *P. mosselii* may probably play a role as shuttles for acquired metallo-β-lactamases resistance thus an antibiogram was made for *P. mosselii* ATCC BAA-99 and MFY161 (see Additional file 1: Table S1). We found that the two strains were resistant towards 6 of the 16 antibiotics tested including the ticarcillin beta-lactam, which could support the above hypothesis.

## Conclusion

In conclusion, our study demonstrates that *P. mosselii* ATCC BAA-99 and MFY161 are cytotoxic towards Caco-2/TC7 cells, have low invasive capacity, induce secretion of human β-defensin 2 (HBD-2), alter the epithelial permeability of differentiated cells and damage the F-actin cytoskeleton. These strains are less virulent than *P. aeruginosa* PAO1, but their behavior resembles that of cytotoxic strains of *P. fluorescens*[17,18] and by thus may be considered as potential emerging human pathogen.

## Methods

### Bacterial strains

*P. mosselii* ATCC BAA-99 is a clinical strain isolated from tracheal aspirate of a patient suffering from pulmonary infections [19]. *P. mosselii* MFY161 was collected from urine of a patient suffering from alcoholic hepatitis in Charles Nicolle hospital (Rouen, France), and characterized by 16SrDNA, *oprF* and *oprD* sequencing [7,8], and siderotyping [22]. *P. aeruginosa* PAO1 was obtained from an international collection. All the strains were routinely cultivated under vigorous shaking, in ordinary nutrient broth (Merk, Darmstadt, Germany), at their optimal growth temperature, 30°C for *P. mosselii* ATCC BAA-99 and MFY161*,* 37°C for *P. aeruginosa* PAO1.

### Cell line and culture

Caco-2/TC7 cells were grown in Dulbecco’s Modified Eagle’s Medium (DMEM, Invitrogen) supplemented with 15% of heat-inactived fetal calf serum, 2 mM of L-glutamine, 100 U.mL^-1^ each of penicillin and streptomycin and 1% of non-essential amino acids. For the experimental assays, the cells were seeded at a density of 10^5^ cells.cm^-2^ in 24-wells tissue culture plates, or on inserts (6.4 mm diameter, 3 μm pore size, Falcon) to obtain fully differentiated cells. The cells were cultured at 37°C in 5% CO_2_-95% air atmosphere and the medium was changed daily.

### Cell infection

Bacteria of overnight culture were harvested by centrifugation (5000 g, 5 min, 20°C) and resuspended at a density of 10^8^ colony forming unit (CFU).mL^-1^ in cell culture medium without serum and antibiotics. Caco-2/TC7 cells grown on 24-wells culture plates or inserts were washed twice with fresh culture medium and the bacterial suspensions were applied to the cell surface at a concentration of 10^8^ CFU.cm^-2^, resulting to a multiplicity of infection (MOI) of 100. Infected cells were then incubated at 37°C in 5% CO_2_-95% air during 24 h for all experiments, excepted 4 h of infection for the invasion test. Each assay was conducted in triplicate in independent experiments (successive passages of Caco-2/TC7 cells).

### Cytotoxicity assay

Cytotoxicity assay was performed on confluent Caco-2/TC7 grown in 24-wells culture plates. After 24 h of infection, the supernatants from Caco-2/TC7 monolayers were collected and the concentration of lactate dehydrogenase (LDH), a cytoplasmic enzyme released upon cell death, was determined using an enzymatic assay (Cytotox 96 Promega, Charbonnieres, France) as previously described [17]. Caco-2/TC7 cells exposed to Triton ×100 (0.9%) were used as a control of total LDH release (100% dead cells).

### Bacterial invasion assay

After 4 h of infection, Caco-2/TC7 monolayers were washed with phosphate-buffered saline (PBS). Adherent bacteria were killed by incubation for 1 h with 300 μg.mL^-1^ gentamycin, an antibiotic that does not cross the cytoplasmic membrane of eukaryotic cells and then only kills bacteria not internalized in cells. Caco-2/TC7 monolayers were washed 3 times with PBS to remove the antibiotic and dead bacteria. The cells were then lysed by incubation for 15 min with 0.5% Triton ×100 to release the intracellular bacteria and the lysates were plated onto nutrient agar to determine the number of internalized bacteria.

### Quantification of IL-6, IL-8 and HBD-2

After 24 h of infection with the bacterial suspensions, the levels of IL-6 and IL-8 cytokines were measured in Caco-2/TC7 cells supernatant using ELISA Quantikine kits (R&D systems). The human β-defensin-2 (HBD-2) was quantified using the Defensin 2, beta (Human) - ELISA Kit (Phoenix Pharmaceuticals inc). These assays were conducted according to the manufacturer’s protocols.

### Transepithelial electrical resistance measurements

Caco-2/TC7 cells grown on inserts were used at 21 days post-confluence (fully differentiated cells) and the transepithelial electrical resistance (TER) of the monolayers infected or not with the bacterial strains was measured during 24 h using the Millicell Electrical Resistance System (Millipore Corp, Bedford, MA). TER values are expressed as percentages of the pre-infection level of the TER (baseline) measured for each individual cell monolayer in the inserts.

### Actin visualisation

Fully differentiated Caco-2/TC7 monolayers were exposed to the bacterial strains for 24 h. At the end of the experiment, the cells were washed with PBS, fixed for 10 min in 3.7% paraformaldehyde and permeabilized for 5 min with 0.1% Triton ×100 at room temperature. The cells were then incubated with 1% bovine serum albumin in PBS for 10 min and the apical F-actin cytoskeleton was stained with Alexa-488 phalloïdin (1U/insert) for 45 min at room temperature. Following three washes in PBS, cell monolayers were examined using a confocal laser scanning microscope (Zeiss, LSM710).

### Statistical analysis

All experiments were conducted independently at least three times. The results are expressed as means +/− SEM and statistical significance were performed by Student’s *t-*test.

## Competing interests

The authors declare that they have no competing interests.

## Authors’ contributions

EB, MF, SC and NC designed the experiments, supervised the research and wrote the paper. CLJ, AM, KB and NC did the experiments and/or data analysis. All authors read and approved the final manuscript.

## Supplementary Material

Additional file 1: Table S1 Antibiotic susceptibility pattern of *P. mosselii* ATCC BAA-99 and *P. mosselii* MFY161. The antibiotics tested were ticarcillin (TIC), piperacillin (PRL),colistin (CT), imipenem (IPM), aztreonam (ATM), tobramycin (TOB), gentamycin (GN), amikacin (AK), ticarcillin + clavulanic acid (TIM), ceftazidime (CAZ), ciprofloxacin (CIP), cefsulodin (CFS), levofloxacin (LEV), trimethoprim-sulphamethoxazole (SXT), fosfomycin (FF) and netilmicine (NET). R, resistant; I, intermediate; S, susceptible. Click here for file
