# Supplementary material for: Cytotoxicity and inflammatory potential of two Pseudomonas mosselii strains isolated from clinical samples of hospitalized patients
Source: BMC Microbiol. 2013 May 29;13:123. doi: 10.1186/1471-2180-13-123 (PMC3679952; doi:10.1186/1471-2180-13-123)
Supplement: Additional file 1: Table S1 — Antibiotic susceptibility pattern of P. mosselii ATCC BAA-99 and P. mosselii MFY161. The antibiotics tested were ticarcillin (TIC), piperacillin (PRL),colistin (CT), imipenem (IPM), aztreonam (ATM), tobramycin (TOB), gentamycin (GN), amikacin (AK), ticarcillin + clavulanic acid (TIM), ceftazidime (CAZ), ciprofloxacin (CIP), cefsulodin (CFS), levofloxacin (LEV), trimethoprim-sulphamethoxazole (SXT), fosfomycin (FF) and netilmicine (NET). R, resistant; I, intermediate; S, susceptible. [file 1471-2180-13-123-S1.pptx]

## Slide 1
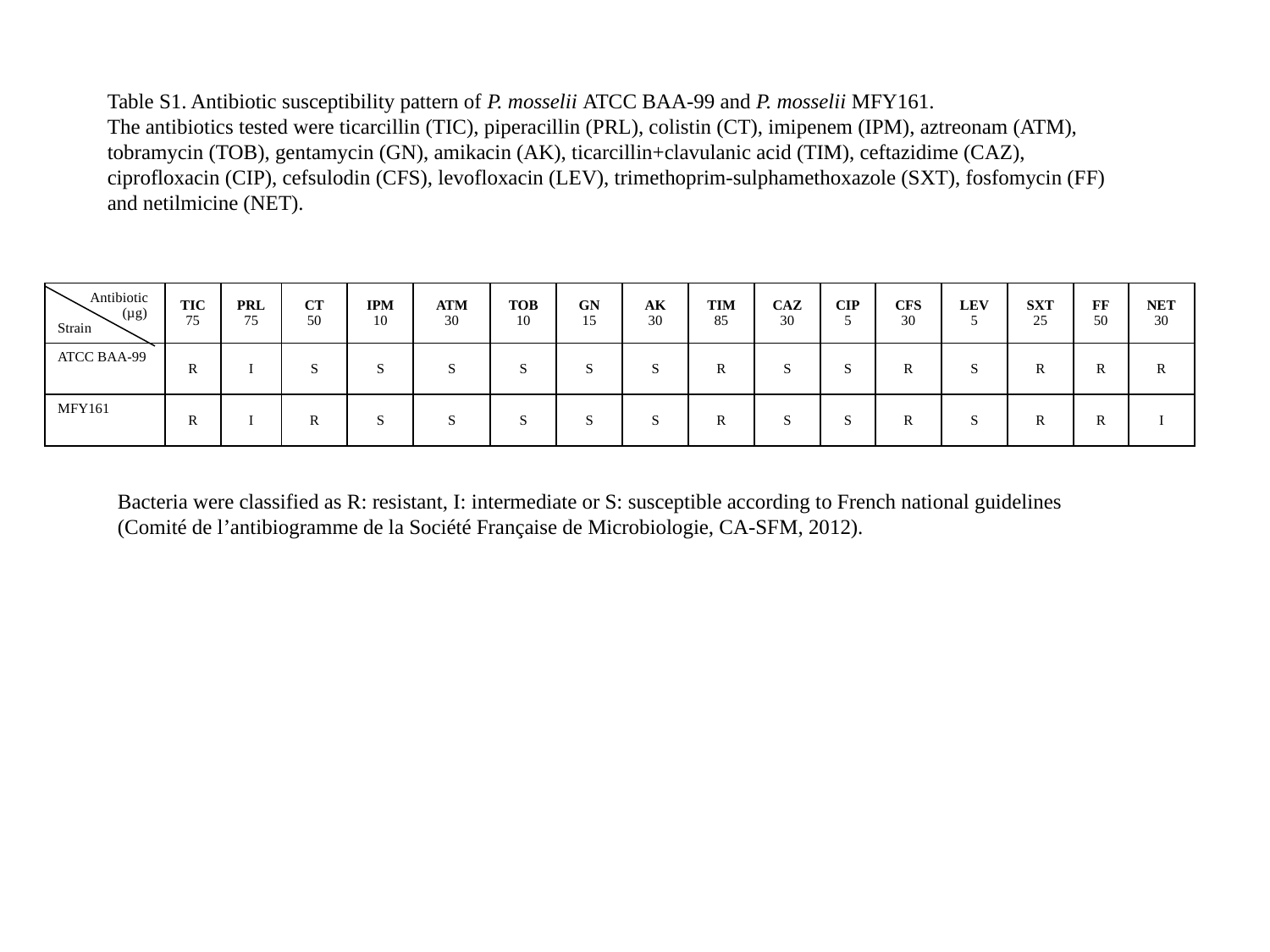

Table S1. Antibiotic susceptibility pattern of P. mosselii ATCC BAA-99 and P. mosselii MFY161.
The antibiotics tested were ticarcillin (TIC), piperacillin (PRL), colistin (CT), imipenem (IPM), aztreonam (ATM), tobramycin (TOB), gentamycin (GN), amikacin (AK), ticarcillin+clavulanic acid (TIM), ceftazidime (CAZ), ciprofloxacin (CIP), cefsulodin (CFS), levofloxacin (LEV), trimethoprim-sulphamethoxazole (SXT), fosfomycin (FF) and netilmicine (NET).
| Antibiotic (µg) Strain | TIC 75 | PRL 75 | CT 50 | IPM 10 | ATM 30 | TOB 10 | GN 15 | AK 30 | TIM 85 | CAZ 30 | CIP 5 | CFS 30 | LEV 5 | SXT 25 | FF 50 | NET 30 |
| --- | --- | --- | --- | --- | --- | --- | --- | --- | --- | --- | --- | --- | --- | --- | --- | --- |
| ATCC BAA-99 | R | I | S | S | S | S | S | S | R | S | S | R | S | R | R | R |
| MFY161 | R | I | R | S | S | S | S | S | R | S | S | R | S | R | R | I |
# Bacteria were classified as R: resistant, I: intermediate or S: susceptible according to French national guidelines (Comité de l’antibiogramme de la Société Française de Microbiologie, CA-SFM, 2012).
